# Cycle length flexibility: is the duration of sexual receptivity associated with changes in social pressures?

**DOI:** 10.1098/rsos.231307

**Published:** 2023-11-29

**Authors:** Fragkiskos Darmis, Élise Huchard, Guy Cowlishaw, Alecia J. Carter

**Affiliations:** ^1^ Department of Anthropology, University College London, London, UK; ^2^ Institut des Sciences de L'Evolution de Montpellier, UMR 5554, CNRS, Université de Montpellier, Montpellier, France; ^3^ Institute of Zoology, Zoological Society of London, London, UK

**Keywords:** cycle length manipulation, female–female competition, sexual coercion, sexual selection, social environment

## Abstract

Research in social mammals has revealed the complexity of strategies females use in response to female-female reproductive competition and sexual conflict. One point at which competition and conflict manifests acutely is during sexual receptivity, indicated by swellings in some primates. Whether females can adjust their sexual receptivity from cycle to cycle to decrease reproductive competition and sexual conflict in response to social pressures has not been tested. As a first step, this study explores whether sexual receptivity duration is predicted by social pressures in wild female chacma baboons (*Papio ursinus*). Given that female baboons face intense reproductive competition and sexual coercion, we predicted that: females could shorten the duration of their sexual receptive period to reduce female–female aggression and male coercion or increase it to access multiple or their preferred male(s). We quantified 157 ovulatory cycles from 46 wild females living in central Namibia recorded over 15 years. We found no support for our hypothesis; however, our analyses revealed a negative correlation between maximal-swelling duration and group size, a proxy of within-group competition. This study provides further evidence that swelling is costly as well as a testable framework for future investigations of ‘cycle length manipulation’.

## 1. Introduction

Recent advances in sexual selection theory underline the importance of female-female competition, along with mate choice, in generating variance in female reproductive success [1–4]. In the first case, females tend to face competition with consexuals over access to males and reproductive resources [5–8]. This competition for access to males increases when there is synchrony of females' fertility (i.e. ovarian synchrony), which creates a female-biased operational sex ratio (OSR) and increases aggression received by receptive females [9,10]. In general, females compete with consexuals for (i) their preferred mates (e.g. chacma baboons *Papio ursinus*: [9]; red-fronted lemurs *Eulemur rufifrons*: [11]), (ii) paternal services for their offspring (e.g. chacma baboons: [12,13]), and (iii) other reproductive resources [14], such as breeding territories (e.g. lek centre [15]) or food resources [16]. Those benefits are achieved either through monopolization of resources directly from females (i.e. contest competition for food) [17] or indirectly (e.g. nutrient-rich spermatophores in insects [18], low predation risk in lek centres [15]). Antagonism with consexuals can manifest through non-aggressive interactions (e.g. signals [18]) and escalate up to physical aggression, which can be detrimental to the receiver's short- or long-term fitness [19].

Considering mate choice, it may be advantageous for females to concentrate paternity into a single male who can provide benefits in the form of good genes or paternal services such as protection to her or her offspring. However, in group living species, a number of social pressures may decrease the possibility for females to express their mating preference. For example, recent studies highlight the role of sexual conflict [20], which frequently manifests via infanticide in mammals. Indeed, infanticide risk may push females to mate with multiple rather than single partners, to dilute paternity certainty among multiple males who subsequently restrain from attacking an offspring that they could have sired [21,22]. Female choice may be further constrained by sexual coercion, when males direct aggression to females [3,20,23], either to increase the probability that they will mate with them or to decrease the probability that they will mate promiscuously [24]. In such cases, females will mate with their aggressors if resisting is more costly than giving in [20]. In groups of water striders (*Aquarius remigis*), aggressive males acquire more matings than less aggressive ones [25]. In primates, male baboons (*Papio ursinus*) that attack pre-ovulatory females are more likely to monopolize them when receptive [26] and females do not copulate close to adult males to avoid bystanders' aggression [26,27]. Also, female chimpanzees (*Pan troglodytes*) initiate copulations most frequently with those males that were most aggressive towards them throughout their cycle [28,29] and male lemurs (*Lemur catta*) that are not preferred can force copulations [30]. However, male coercion can lead to female counterstrategies and antagonistic co-evolution between the sexes [20] since coercion is associated with multiple costs for females. For example, the social dynamics involving male immigration events, high male-male competition and unstable male hierarchies are stressful for females because they typically increase the intensity of coercion [13,31,32] and harassment [33–35]. Moreover, females suffer costs from coercion when: they are forced to mate with subordinate or incompatible males; direct aggression or harassment increases stress hormone secretion [29]; males reduce their preferred level of parental care [23]; or coercion impairs female choice [31].

By decreasing the length of their receptive period, females could potentially decrease (i) time that they are subject to aggression they receive from other females (female-female competition), (ii) and/or males (sexual coercion). By contrast, by increasing the length of their receptive period, females could potentially (iii) increase their probability of mating with a preferred male (female choice), or (iv) multiple males (paternity confusion). Evidence for manipulation of female cycles exists [19,35–37], with studies detecting physiological changes in female cycling patterns (e.g. changes to cycle length, resumption of cycling) as stress responses to social pressures. Socially-stressed primate females, for example by being harassed near menstruation, have longer menstrual cycles [35]. Cycling female yellow baboons (*Papio cynocephalus*) that receive aggression from consexuals tend to have more cycles to conception and longer interbirth intervals [19], while pregnant females that are systematically harassed experience reduced fitness (through abnormal gestation length, spontaneous abortion, and premature delivery) [19]. Psychosocial stress stemming from social instability, in the form of male take-overs, also induces female physiological responses: the presence of unfamiliar males can mediate early sexual maturation [38,39] or abortion [40,41] in female mammals, in a likely effort to limit infanticide risk. Novel males in geladas (*Theropithecus gelada*) prompt females of any reproductive state (immature, cycling, lactating, or pregnant) to start or resume cycling [39,42], while female hamadryas (*Papio hamadryas*) with young infants develop swellings to advertise a reduced postpartum amenorrhea that results in copulations with novel males during take-overs [37]. However, these swellings are deceptive signals that ultimately decrease infanticide risk (four out of five infants survived in [37]) since females did not conceive during the first cycles after the take-over, their reproduction was not accelerated and the interbirth intervals were not shorter compared to times with no male take-overs. Despite the above results, to our knowledge no study to date has proposed a theoretical framework that considers cycle length manipulation as a counterstrategy to sexual selection pressures. Additionally, the aforementioned studies provide evidence of cycle manipulation as an acute reaction to social pressures but have not explicitly tested or explored fluctuations in sexual receptivity in response to changes in females’ social pressures under a proximate framework.

In this study, we explored whether there is a correlation between females' oestrous duration and social pressures. We assumed that females could adjust their oestrous cycles to either reduce aggression from (i) coercive males or (ii) females, or to increase their access to (iii) their preferred or (iv) multiple males. We focused on female chacma baboons because they exhibit elevated female–female competition compared to other primates [13,43,44], live in multimale-multifemale groups, mate promiscuously [26,45], and have exaggerated swellings that reliably indicate females’ stage of the follicular phase of the oestrus cycle [36]. Female chacma baboons are an ideal model to study female-female competition, intersexual competition, as well as sexual coercion, since they target the dominant male as a sexual partner [13,44] but also mate promiscuously [9] outside their peak swelling to establish more males with a non-zero paternity probability or to initiate male–male competition (e.g. sperm competition) [27,36]. In addition, males' monopolization of only one receptive female at a time (i.e. mate-guarding: [7,12]) results in female-female competition over sexual access to particular males [9,13], who might be better able to protect them from aggressive consexuals or provide future postpartum care for the offspring [46].

To address our overarching aim, we proceeded in two steps. First, we explored the variation within and among females in the durations of swelling (SD) and maximal-swelling duration (MSD) stages of the cycle and we predicted that different mechanisms could act on each part of the cycle. We predicted that there would be more variation within females’ cycles than between females across each of their cycles. Second, we tested whether variation in the duration of specific cycle phases was associated with social pressures (summarized in table 1).

**Table 1.** Hypotheses, predictions and support for the hypotheses proposed to affect the duration of female cycle-specific phases.

hypotheses		predictions	support?^a^	
hypothesis	explanation		SD^b^	MSD^c^
H1: intrasexual selection	H1: *avoidance of female aggression hypothesis* = pregnant and lactating females predict shorter durations of female sexual receptivity, possibly because those females could attempt to decrease their exposure to aggression	↑ P females + L females^d^ → ↓ SD to ↓ consexual aggression	—	—
		↑ PL^e^ → ↓ MSD to ↓ consexual aggression		
H2: intersexual selection and sexual conflict	H2a: *paternity concentration hypothesis* = female sexual receptivity is predicted to increase when several other females are simultaneously swollen, possibly because this increase could correlate with higher chances of mating with the alpha male	↑ simultaneously receptive females → ↑ (M)SD to ↑ access to preferred (dominant) male	­—	—
	H2b: *paternity confusion hypothesis* = female sexual receptivity is predicted to increase in response to a female-biased operational sex-ratio, possibly because females attempt to mate with multiple males and decrease infanticide risk	female-biased OSR → ↑ (M)SD to mate with many males and confuse paternity	—	—
	H2c: *avoidance of male coercion hypothesis =* female sexual receptivity is predicted to decrease in response to strong male-male competition and social instability, possibly because females attempt to decrease their exposure to sexual coercion. Strong male-male competition and social instability can be inferred by an increasing number of males	male-biased OSR or ↑ males → ↓ (M)SD to avoid coercive behaviour	males: —	males: —
			OSR: —	OSR: —

^a^— = no support.

^b^SD = swelling duration.

^c^MSD = maximal-swelling duration.

^d^PL = the number of pregnant and of lactating females.

^e^The sum of pregnant and lactating.

First, considering female intrasexual selection, we tested whether female competition for paternal care was associated with variation in the duration of receptivity (the avoidance of female aggression hypothesis (H1). Pregnant and lactating females aggressively target swollen females, which reduces their likelihood of conception, to decrease competition for paternal care of their own offspring, or to induce reproductive suppression so that their own infants are not born at the same time as many others [33]. For this reason, we predicted that a higher number of pregnant and lactating females would translate into receptive females terminating the swelling earlier and, ultimately, decreasing their exposure to consexual aggression.

Our second hypothesis dealt with intersexual selection and sexual conflict (H2): we hypothesized that females' SD and MSD would show a correlation with competition for access to the dominant mate (the paternity concentration hypothesis, H2a). We predicted that, when competition for access to the alpha male was likely because there were simultaneously swollen individuals, females would respond by delaying ovulation—through an elongation of their swelling or MSD—to increase the probability of mating with this male. By doing so females may benefit from (i) good genes [13,36] and (ii) concentrating paternity certainty in their preferred male, usually the dominant, who is also the most likely to pose a future risk of infanticide if they fail to mate with him [13,47].

We also tested whether the risk of infanticide from multiple males correlated with patterns of variation in female baboons’ cycle-phases (the paternity confusion hypothesis, H2b). We predicted that an increase in SD and MSD will be associated with a female-skewed OSR so that females can mate with many males to reduce infanticide risk. Finally, we tested whether the effect of social instability in the form of high numbers of males (i.e. strong male–male competition) correlated with patterns of variation in a female's receptivity phases. We predicted that a male-skewed OSR or a high number of males translates into high male–male competition, which in turn increases the risk of sexual coercion and male-initiated aggression for receptive females [12,26]. Under this scenario, a negative association between oestrous duration and the number of males or a male-biased OSR might provide evidence for females' attempts to escape or minimize the aggression received from coercive males (the avoidance of male coercion hypothesis, H2c)**.**

## 2. Material and methods

### 2.1. Study site and animals

Data were collected from all adult females within two habituated groups, J and L, of a wild chacma baboon population at Tsaobis Leopard Park (22°22′ S, 15°44′ E) in central Namibia. Studies on this population, which have been ongoing since 2000 [48], have revealed that the naturally foraging baboons [48] exhibit low predation risk [49] and high female–female aggression rates [9,13] that can result in reproductive suppression [13]. Over the course of the study, troop numbers fluctuated around a median of 55 (44–69) in J troop and 52 (21–71) in L troop.

The data used in this study were collected periodically over 15 years, from 2005 to 2019, during field seasons lasting between two and eight months per annum, usually during the austral winter. During field seasons, troops were visited daily, when possible, with some interruptions for other tasks or unforeseen circumstances. During troop visits, we collected data on troop composition (see below) and individual behaviour ad libitum.

Individuals’ ages were known from observing births or from patterns of dentition wear following capture. Individuals' relative ranks were calculated annually from ad libitum recorded dominance interactions in the package Matman 1.1.4 (Noldus Information Technology, 2013). Absolute ranks were converted to a relative scale ranging from 0 (lowest rank) to 1 (highest rank), to control for group size, using the formula 1 − ((1 − *r*)/(1 − *n*)), with *r* being individual's absolute rank ranging from 1 to the total group size *n*. Group size was calculated as the total number of individuals in the troop for a particular year. Individuals who were present for less than half of a field season (owing to emigration or death) were not included.

### 2.2. Troop composition and reproductive state data

Each day that the troop was contacted, a census was completed that recorded the identities of the individuals present and the reproductive states of each adult female. Females' reproductive states were recorded as: (i) pregnant (determined *post hoc* based on lack of resumption of swelling, reddening of the paracallosal skin and subsequent birth); (ii) lactating (i.e. period following the birth of an infant until cycle resumption); (iii) oestrous/swollen (i.e. exhibiting periovulatory swelling of the anogenital region); or (iv) non-swollen (i.e. deturgescent but not pregnant). For females in oestrus, we also recorded swelling sizes on a semi-quantitative scale from 0 (smallest) to 4 (largest), in order to capture within-individual variation in swelling size across successful oestrus cycles [50]. As mentioned above, baboon troops were not followed daily, and as such there were some missing data during some females’ cycles. We discarded any observations of the entire oestrous cycle where the start or end of the SD was missing and thus could introduce inaccuracy.

For females in oestrus, SD was calculated as the number of continuous days that a female was recorded with a swelling of any size. Because different mechanisms could act on the different parts of the cycle, in addition to SDs, we calculated the MSD as the number of days the focal female exhibited the largest swelling size during that respective cycle. In summary, we used 157 receptivity observations for SD from 46 females (median number of swellings/female = 5, range = 1–14) and 150 receptivity observations for MSD (median number of swellings/female = 5, range = 1–14). The difference between the number of observations of these two variables stems from the fact that for seven observations of SD the start and end date of receptivity were accurately known, yet this was not the case for the MSD of those cycles.

We calculated seven predictor variables describing social pressures females potentially face: to consider (H1) female-female competition for paternal care, we calculated (i) the number of pregnant and (ii) of lactating females, as well as (iii) their sum (pregnant and lactating (PL)), during the time a focal female started swelling. To consider (H2a) competition for females' access to the preferred male, for the first day of a focal female's swelling we calculated the number of simultaneously swollen females that were (iv) not maximally-swollen and (v) maximally-swollen. To consider sexual conflict in terms of (H2b) infanticide risk and (H2c) sexual coercion, we calculated the (vi) OSR as the ratio of sexually active females (i.e. swollen at any level) to adult males and (vii) the number of adult males.

### 2.3. Statistical analyses

All statistical analyses were performed using R (v. 4.0.3, 2020-10-10). Data and R code are available on Dryad: https://doi.org/10.5061/dryad.r4xgxd2h3 [51].

### 2.4. Question 1: repeatability of receptivity

To determine how much variation in females' receptivity part of the cycle was owing to differences between individuals, we calculated the repeatability (*R*) of the SD and MSD of all the females that had at least two measurements for each response (*n* = 38). *R* is calculated as $\sigma_{\alpha }^{2}/\sigma_{\alpha }^{2}+\;\sigma_{\varepsilon }^{2}$, where $\sigma_{\alpha }^{2}$ represents the between-group variance, $\sigma_{\varepsilon }^{2}$ the within-group variance ($\sigma_{\alpha }^{2}+\;\sigma_{\varepsilon }^{2}=\;$ the total phenotypic variance, $V_{p}$ [52]); in this case, the ‘group’ is an individual female. A relatively high *R* estimate would indicate that the dependent variable exhibits high between- and low within-female variance; low values of *R* indicate traits with high within-female or low between-female variation [52]. We calculated *R* using the rpt function (rptR package), which can calculate repeatability for Poisson-distributed data, and the models included female identity and troop as random effects. Finally, both models controlled for the possible confounding fixed effects [53] of age, rank, and, because larger groups could result in increased competition for limited resources (i.e. feeding competition), which could impact females' condition and their SD [7], we also controlled for group size—the number of adults and sub-adults present in the troop. The permutations and bootstrapping were set to 1000 and confidence intervals (CIs) at 95%. However, we are aware that the repeatability we present in the results might be somewhat flawed as MSD is overdispersed (the rptR package offers no way to correct for that excess variance though).

### 2.5. Question 2: social determinants of oestrous length

To determine whether social pressures were correlated with females’ fluctuations in sexual receptivity duration, we used two generalized linear mixed models (GLMMs; glmer function, lme4 package [54]) with the response variables: SD and MSD. SD was modelled using a Poisson distribution and MSD using a negative binomial distribution to correct for overdispersion. Female identity nested in troop identity was included as a random effect for SD models and female identity was a random effect for MSD models, since variation between troops was absent for MSD. In both models, we included rank, age and group size as control variables for the same reasons as we did when estimating repeatability.

We determined, with the use of Akaike information criterion corrected for small sample size (AICc), which predictors should be used to test for the effect of pregnant and lactating consexuals on sexual receptivity duration (H1) as we did not have an *a priori* prediction of whether the number of pregnant and of lactating females or the combined number (i.e. sum) of PL females would be a better predictor. The results of model selection led us to include pregnant and lactating females separately for SD and the sum for MSD (electronic supplementary material, table SA1). In both SD and MSD models, we included an interaction with rank because rank usually determines who can harass whom. To explore the effect of the number of receptive females on sexual receptivity duration (H2a), we used the number of not maximally swollen females when a focal female started swelling when the response was SD; and the number of maximally swollen females when a focal female started swelling when the response was MSD. Thirdly, to determine whether the risk of infanticide (H2b) or male-male competition (part of H2c) predicted fluctuations in female sexual receptivity durations, we included the OSR as a predictor. Lastly, to determine the response of sexual receptivity durations to male-male competition (part of H2c), we included the number of adult males as a predictor because it is a proxy of social instability in the sense that male ranks are more volatile when there is more competition (i.e. more males) (A.J. Carter, E. Huchard 2006–2023, personal observation).

To control for possible type I errors [55], we then tested whether a model with random slopes for each of our predictors performed better than one without (electronic supplementary material, tables SA2 and SA3), using AICc, to compare models (aictab function, AICcmodavg package [56]). We included this analysis as some authors argue that random slopes are crucial to avoid false positive rates [57,58]. The best-fit model for SD and MSD, carrying 92% and 69% of the cumulative model weight (AICcWt), respectively, and showing the lowest AICc values, did not include random slopes. For the two resulting models we checked for multicollinearity of model terms using variance inflation factors (VIFs; electronic supplementary material, figures SA2 and SA4, respectively; check_collinearity function, performance package [59], a VIF < 5 indicates a low correlation of the predictor with other predictors and a VIF > 10 a high correlation [60]). All VIFs were less than 5, except interaction terms, which is expected [61], so these variables were retained. Lastly, we performed full-null model comparisons using a likelihood-ratio test. The null models contained only the control variables age, relative rank, group size and, as random factors, female identity (ID) nested in troop for SD and female ID for MSD (see above). For SD, the full model performed better than the null model (electronic supplementary material, table SA4), but for MSD this was not the case. Therefore, we rejected our hypotheses for MSD and we report the results of the null model that contained age, rank and group size as covariates. Residuals and quantile-quantile plots for the retained models were also checked for normality (for model diagnostics see the electronic supplementary material, figures SA1–SA4).

## 3. Results

Females were swollen for an average (median) of 22 days (range 8–44) and maximally-swollen for a median of 6 days (range 1–24). There were no differences between troops (Mann-Whitney *U*-test: SD, *W* = 3481.5, *p* = 0.10; MSD, *W* = 2651.5, *p* = 0.59).

### 3.1. Question 1: repeatability of receptivity

Neither SD nor MSD were repeatable at the individual level (SD: *R* = 0.038, s.e. = 0.052, CI = [0, 0.184], *p* > 0.05; MSD: *R* = 0, s.e. = 0.038, CI = [0, 0.126], *p* > 0.05) (electronic supplementary material, table SA5 and figure SA5).

### 3.2. Question 2: social determinants of swelling duration and maximal-swelling duration

**Figure 1.**
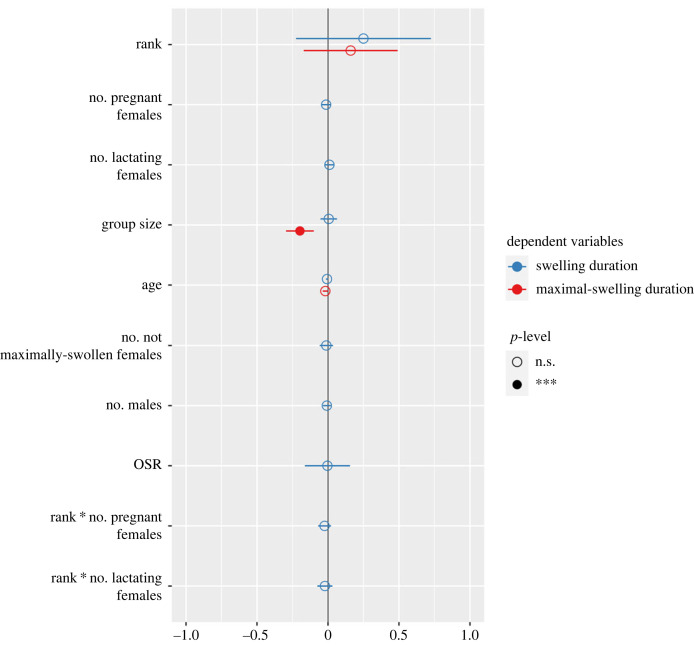
Forest plot of standardized estimates of the models with swelling duration (red) and maximal-swelling duration (MSD) (the null model is shown; blue) as response variables. The level of significance is indicated by an unfilled circle (*p* > 0.05) or a filled circle (*p* < 0.001). Only group size significantly predicted a decreased MSD in the null model.

**Figure 2.**
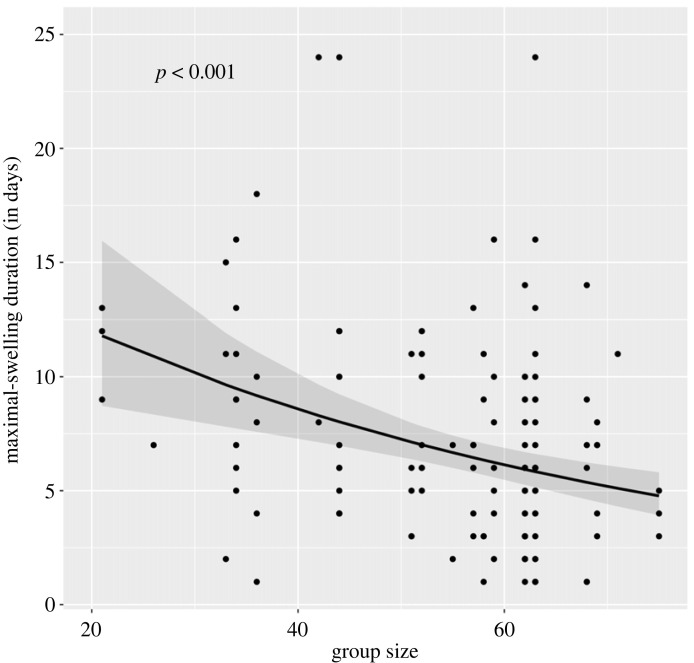
The effect of group size on maximal-swelling duration. The line indicates the predicted effect by varying the respective focal variable and by setting all the other covariates to their mean. The shaded area represents the 95% CI.

We included three control variables in both our models (table 2). Rank was not significantly associated with either of our responses (SD: *β* = 0.25, s.e. = 0.24, *p* > 0.05; MSD: *β* = 0.16, s.e. = 0.17, *p* > 0.05; figure 1), and neither was age (for SD, *β* = −0.01, s.e. = 0.004, *p* = 0.09; for MSD, *β* = −0.02, s.e. = 0.01, *p* = 0.051; figure 1). Group size also did not correlate with SD (*β* = 0.01, s.e. = 0.03, *p* > 0.05), but it did for MSD (i.e. the null model), that is predicted a decrease in MSD (*β* = −0.20, s.e. = 0.05, *p* < 0.001; figures 1 and 2; table 2). Specifically, a one-unit increase in group size negatively correlated with a drop of almost 18% in MSD.

**Table 2.** Results of the full model with SD and the null model with MSD as response variables. (‘Log-mean’ measures the effect of the specific predictor on the response variable and is given in log-scale. ‘s.e.’ indicates the standard error and CI shows the 95% confidence intervals. Significant results are indicated in bold.)

coefficients	model 1 (SD)				model 2 (MSD)			
	log-mean of estimates	s.e.	CI (95%)	*p*-value	log-mean of estimates	s.e.	CI (95%)	*p*-value
intercept	3.30	0.22	2.88–3.72	<0.001	2.02	0.15	1.73–2.32	<0.001
rank	0.25	0.24	−0.22–0.72	0.302	0.16	0.17	−0.17–0.49	0.344
rank*number of pregnant females	−0.02	0.02	−0.07–0.02	0.296	—	—	—	—
rank*number of lactating females	−0.02	0.03	−0.07–0.03	0.424	—	—	—	—
number of lactating females	0.01	0.02	−0.02–0.05	0.574	—	—	—	—
number of pregnant females	−0.01	0.02	−0.04–0.02	0.393	—	—	—	—
age	−0.007	0.004	−0.02–0.001	0.09	−0.02	0.01	−0.04–0.0001	0.051
number of not maximally-swollen females	−0.01	0.02	−0.06–0.04	0.622	—	—	—	—
number of males	−0.01	0.01	−0.04–0.02	0.566	—	—	—	—
OSR (females/males)	−0.004	0.08	−0.16–0.15	0.960	—	—	—	—
group size	0.01	0.03	−0.05–0.06	0.858	−0.20	0.05	−0.30–−0.10	<0.001

#### 3.2.1. H1: intrasexual selection

##### 3.2.1.1. Avoidance of female aggression hypothesis

Under our first hypothesis, we tested whether the number of pregnant and lactating females was associated negatively with females' SD or MSD. There were on average 10 (median, range = 3–28) PL females, five pregnant (median, range = 0–11) as well as six lactating (median, range = 0–18) during females’ swelling and maximal-swelling phase. There was no relationship between the latter two variables and SD (pregnant: *β* = −0.01, s.e. = 0.02, *p* > 0.05; lactating: *β* = 0.01, s.e. = 0.02, *p* > 0.05) and there was also no effect of the interaction of these variables with rank. For MSD, PL was not a predictor that improved model fit as the full-null model comparison was non-significant.

#### 3.2.2. H2: intersexual selection and sexual conflict

##### 3.2.2.1. H2a: paternity concentration hypothesis

To test our second hypothesis, we explored whether there was positive association between SD and MSD and the number of swollen females. There were on average two (median, range = 0–6) not maximally-swollen females and 0 (median, range = 0–3) maximally-swollen females when a focal female started swelling. Neither response was associated with swelling durations D, not-maximally-swollen females: *β* = −0.01, s.e. = 0.02, *p* > 0.05; MSD, non-significant full-null model comparison; table 2; figure 1).

##### 3.2.2.2. H2b: paternity confusion hypothesis

We predicted a positive correlation between females' SD or MSD and a female-biased OSR. The mean OSR value was 0.64 (range 0.1–2.5): on average there were more sexually active males than females. Our analyses showed no correlation between OSR and SD (*β* = 0.003, s.e. = 0.09, *p* > 0.05; figure 1) and, again, the insignificant full-null model comparison strongly suggested against OSR predicting variation in MSD.

##### 3.2.2.3. H2c: avoidance of male coercion hypothesis

We tested whether there was a negative correlation between the number of males and receptivity durations. There were on average five males (median, range = 1–10) when females started to swell. We found no support for this hypothesis either (SD: *β* = −0.01, s.e. = 0.02, *p* > 0.05; MSD: found not to be a good predictor; figure 1; table 2).

## 4. Discussion

The present study investigated the variation in the length of receptivity within versus across females. We provided a theoretical framework for why females could evolve to manipulate their receptivity—a mechanism we termed cycle length manipulation. We further tested whether variation in female sexual receptivity could be associated with changes in social pressures. We detected high within-female variation in the length of sexual receptivity. However, we found no evidence that variation in females’ sexual receptivity is strategically adjusted to decrease intra- or inter-sexual competition. We did, however, find that group size, and presumably within-group competition, negatively affected females' maximal swelling durations.

We found that SD and MSD were not repeatable. Our results do not mean that there are no between-individual differences in SD and MSD, but that those differences are not large relative to within-individual differences and not consistent between females. Our findings show that cycle phases are labile traits that females could potentially manipulate. At a comparative level, high variation in oestrous duration has been detected in animals (e.g. captive jaguars, with a mean duration of 10.42 and a range of 7 to 15 days [62]). Women, in contrast, show repeatable cycle lengths, even though there is some variation within [63] and across women [64], as well as extensive variation in average cycle length across human populations [65]. It may be that baboons’ low repeatability of receptivity reflects the harsh environmental conditions at Tsaobis, with females' condition fluctuating in response.

We found no evidence that the numbers of pregnant and lactating consexuals correlated with receptivity duration. One reason why our predictor did not affect receptivity duration could be that female reproductive suppression in the form of aggression was given by the female friends of a particular male [12]. Specifically, aggression was initiated by pregnant and lactating females who were friends of the male that was mate-guarding the receptive female. A more targeted approach that considers as a predictor the number of female friends a swollen female's mate-guarding male has might provide evidence for cycle length manipulation. Alternatively, it may be that the particular stage of lactation and pregnancy might be a better predictor than just the total number of individuals in each phase. For example, a study in yellow baboons revealed an increasing attack rate initiated from lactating females as lactation stages progressed [19].

We predicted that higher numbers of receptive females would prompt an elongation of oestrous duration, so that females could concentrate paternity in the alpha male but found no support for this. At least two explanations may account for this absence of evidence. First, because female chacma baboons are non-seasonal breeders [13], oestrous synchrony in this population is low, with a median of two simultaneously swollen females (i.e. swollen at any phase of their cycle). Consequently, mating with the preferred male is possible for most cycling females and therefore elongation of the fertile period is not necessary. Second, the substantial aggression among cycling females in Tsaobis may limit females' propensity to extend their receptivity period [9,13]. This is predominantly true for lower-ranking oestrous females and females who are not mate-guarded, which tend to be the usual target of aggression from higher-ranking females.

We found no support for hypothesis H2b, that there would be a positive association between an OSR with receptivity durations. Our results could suggest that paternity dilution through extended receptivity comes at a cost for females, which we believe is an increased rate of female-female competition and aggression. Under this scenario, it seems that females entering the mating pool are primarily aggressive towards consexuals at peak swelling, which are most likely monopolized by the dominant male.

The number of adult males, as well as the OSR, were not associated with receptivity durations for SD and were not a good predictor for MSD, as suggested by hypothesis H2c. While male aggression, intimidation and coercion are costly for females in this population [26,27], it could be that a decrease in the length of swelling might incur higher costs, such as a smaller swelling, which could lead to a decrease in the likelihood that higher-ranking males mate-guard. Alternatively, selection to elongate receptivity owing to increased infanticide risk (seen at a comparative level across Cercopithecine primates [36]) but also to decrease receptivity to avoid coercion could result in no effect overall.

We found that group size correlated negatively with MSD when we considered the null model, however the same was true when looking at the full model (results not shown). There are at least two possible explanations for this relationship that are not mutually exclusive and could operate simultaneously. Firstly, as group size increases, competition for limited resources intensifies and maintaining energetically costly maximal swellings could become more difficult for females [7]. In addition, larger baboon groups tend to travel longer distances over greater areas, which could generate higher energetic demands [66]. This interpretation recognizes the energetic limitation that arises owing to elevated levels of feeding competition. Secondly, in baboons, socially induced stress may generate higher cortisol levels [67] and subsequently result in a decrease of the swollen period [33,35]. As the number of in-group individuals increases, receptive females might be subject to higher aggression rates, both from consexuals (intrasexual aggression) and heterosexuals (intersexual aggression). Female primates of larger groups tend to show higher faecal glucocorticoid concentrations [66,68], which could decrease females’ receptivity durations indirectly. Under this scenario, social stress is interlinked with social competition, and future studies can directly test for that by including hormonal data and behavioural observations. Group size, however, was not associated with SD. This might be because (i) SD is comparatively less costly to maintain, even under stress, compared to MSD; or/and (ii) competition with females and males is comparatively less during the whole SD compared to MSD.

In summary, this study correlated variation in the length of female sexual receptivity with social pressures that may influence the intensity of female reproductive competition and intersexual conflict. We found no support for sexual conflict or female-female competition driving receptivity duration. We did, however, find that group size—and presumably within-group competition—correlated with MSD in females. This suggests that maintaining large swellings is costly for females. Further investigations may benefit from including behavioural and hormonal data in order to integrate more closely the proximate mechanisms with ultimate explanations when testing this or related hypotheses.

## Data Availability

Data are available from the Dryad Digital Repository: https://doi.org/10.5061/dryad.r4xgxd2h3 [51]. Data are also provided in the electronic supplementary material [69].

## References

[1] Kleindorfer S, Wasser SK. 2004 Infant handling and mortality in yellow baboons (*Papio cynocephalus*): evidence for female reproductive competition? Behav. Ecol. Sociobiol. **56**, 328-337. (10.1007/s00265-004-0798-1)

[2] Drea CM. 2005 Bateman revisited: the reproductive tactics of female primates. Integr. Comp. Biol. **45**, 915-923. (10.1093/icb/45.5.915) 21676842

[3] Stumpf RM, Martinez-Mota R, Milich KM, Righini N, Shattuck MR. 2011 Sexual conflict in primates. Evol. Anthropol. **20**, 62-75. (10.1002/evan.20297) 22034105

[4] Tang-Martínez Z. 2016 Rethinking Bateman's principles: challenging persistent myths of sexually reluctant females and promiscuous males. J. Sex Res. **53**, 532-559. (10.1080/00224499.2016.1150938) 27074147

[5] Clutton-Brock T. 2007 Sexual selection in males and females. Science **318**, 1882-1885. (10.1126/science.1133311) 18096798

[6] Clutton-Brock T. 2009 Sexual selection in females. Anim. Behav. **77**, 3-11. (10.1016/j.anbehav.2008.08.026)

[7] Huchard E, Courtiol A, Benavides JA, Knapp LA, Raymond M, Cowlishaw G. 2009 Can fertility signals lead to quality signals? Insights from the evolution of primate sexual swellings. Proc. R. Soc. B **276**, 1889-1897. (10.1098/rspb.2008.1923) PMC2674499 19324772

[8] Vaillancourt T. 2013 Do human females use indirect aggression as an intrasexual competition strategy? Phil. Trans. R. Soc. B **368**, 20130080. (10.1098/rstb.2013.0080) 24167310 PMC3826209

[9] Huchard E, Cowlishaw G. 2011 Female–female aggression around mating: an extra cost of sociality in a multimale primate society. Behav. Ecol. **22**, 1003-1011. (10.1093/beheco/arr083)

[10] Tobler R, Pledger S, Linklater W. 2010 No evidence for ovarian synchrony or asynchrony in hamadryas baboons. Anim. Behav. **80**, 829-837. (10.1016/j.anbehav.2010.07.018)

[11] Kappeler PM, Fichtel C. 2012 Female reproductive competition in *Eulemur rufifrons*: eviction and reproductive restraint in a plurally breeding Malagasy primate. Mol. Ecol. **21**, 685-698. (10.1111/j.1365-294X.2011.05255.x) 21880091

[12] Baniel A, Cowlishaw G, Huchard E. 2018 Jealous females? Female competition and reproductive suppression in a wild promiscuous primate. Proc. R. Soc. B **285**, 20181332. (10.1098/rspb.2018.1332) PMC6158522 30185648

[13] Baniel A, Cowlishaw G, Huchard E. 2018 Context dependence of female reproductive competition in wild chacma baboons. Anim. Behav. **139**, 37-49. (10.1016/j.anbehav.2018.03.001)

[14] Watson NL, Simmons LW. 2010 Reproductive competition promotes the evolution of female weaponry. Proc. R. Soc. B **277**, 2035-2040. (10.1098/rspb.2009.2335) PMC2880095 20200030

[15] Bro-Jørgensen J. 2002 Overt female mate competition and preference for central males in a lekking antelope. Proc. Natl Acad. Sci. USA **99**, 9290-9293. (10.1073/pnas.142125899) 12089329 PMC123133

[16] Robinson MR, Kruuk LEB. 2007 Function of weaponry in females: the use of horns in intrasexual competition for resources in female Soay sheep. Biol. Lett. **3**, 651-654. (10.1098/rsbl.2007.0278) 17711817 PMC2121329

[17] Stockley P, Bro-Jørgensen J. 2011 Female competition and its evolutionary consequences in mammals. Biol. Rev. Camb. Philos. Soc. **86**, 341-366. (10.1111/j.1469-185X.2010.00149.x) 20636474

[18] Rosvall KA. 2011 Intrasexual competition in females: evidence for sexual selection? Behav. Ecol. **22**, 1131-1140. (10.1093/beheco/arr106) 22479137 PMC3199163

[19] Wasser SK, Starling AK. 1988 Proximate and ultimate causes of reproductive suppression among female yellow baboons at Mikumi National Park, Tanzania. Am. J. Primatol. **16**, 97-121. (10.1002/ajp.1350160202) 31968869

[20] Watson-Capps JJ. 2009 2 Evolution of sexual coercion with respect to sexual selection and sexual conflict theory. In Sexual coercion in primates and humans (eds MN Muller, RW Wrangham), pp. 23-41. Cambridge, MA: Harvard University Press. (10.4159/9780674054349-002.

[21] van Schaik CP, Pradhan GR, van Noordwijk MA. 2004 Mating conflict in primates: infanticide, sexual harassment and female sexuality. In Sexual selection in primates: new and comparative perspectives (eds CP van Schaik, PM Kappeler), pp. 131-150. Cambridge, UK: Cambridge University Press. (10.1017/CBO9780511542459.010)

[22] Qi X-G, Grueter CC, Fang G, Huang P-Z, Zhang J, Duan Y-M, Huang Z-P, Garber PA, Li B-G. 2020 Multilevel societies facilitate infanticide avoidance through increased extrapair matings. Anim. Behav. **161**, 127-137. (10.1016/j.anbehav.2019.12.014)

[23] Clutton-Brock TH, Parker GA. 1995 Sexual coercion in animal societies. Anim. Behav. **49**, 1345-1365. (10.1006/anbe.1995.0166)

[24] Stumpf RM, Boesch C. 2010 Male aggression and sexual coercion in wild West African chimpanzees, *Pan troglodytes verus*. Anim. Behav. **79**, 333-342. (10.1016/j.anbehav.2009.11.008)

[25] Eldakar OT, Gallup AC. 2011 The group-level consequences of sexual conflict in multigroup populations. PLOS ONE **6**, e26451. (10.1371/journal.pone.0026451) 22039491 PMC3200328

[26] Baniel A, Cowlishaw G, Huchard E. 2017 Male violence and sexual intimidation in a wild primate society. Curr. Biol. **27**, 2163-2168.e3. (10.1016/j.cub.2017.06.013) 28690113

[27] Baniel A, Delaunay A, Cowlishaw G, Huchard E. 2019 Oestrous females avoid mating in front of adult male bystanders in wild chacma baboons. R. Soc. Open Sci. **6**, 181009. (10.1098/rsos.181009) 30800354 PMC6366197

[28] Muller MN, Thompson ME, Kahlenberg SM, Wrangham RW. 2011 Sexual coercion by male chimpanzees shows that female choice may be more apparent than real. Behav. Ecol. Sociobiol. **65**, 921-933. (10.1007/s00265-010-1093-y)

[29] Muller MN, Kahlenberg SM, Emery Thompson M, Wrangham RW. 2007 Male coercion and the costs of promiscuous mating for female chimpanzees. Proc. R. Soc. B **274**, 1009-1014. (10.1098/rspb.2006.0206) PMC2141672 17264062

[30] Parga JA, Henry AR. 2008 Male aggression during mating: evidence for sexual coercion in a female dominant primate? Am. J. Primatol. **70**, 1187-1190. (10.1002/ajp.20609) 18702079

[31] Polo P, Hernández-Lloreda V, Colmenares F. 2014 Male takeovers are reproductively costly to females in hamadryas baboons: a test of the sexual coercion hypothesis. PLoS ONE **9**, e90996. (10.1371/journal.pone.0090996) 24621865 PMC3951309

[32] Beehner JC, Bergman TJ, Cheney DL, Seyfarth RM, Whitten PL. 2005 The effect of new alpha males on female stress in free-ranging baboons. Anim. Behav. **69**, 1211-1221. (10.1016/j.anbehav.2004.08.014)

[33] Wasser S. 1983 Reproductive competition and cooperation among female yellow baboons. In Social behavior of female vertebrates (ed. SK Wasser), pp. 349-390. New York, NY: Academic Press. (10.1016/B978-0-12-735950-2.50018-9)

[34] Dunbar RIM, Sharman M. 1983 Female competition for access to males affects birth rate in baboons. Behav. Ecol. Sociobiol. **13**, 157-159.

[35] Rowell TE. 1970 Baboon menstrual cycles affected by social environment. J. Reprod. Fertil. **21**, 133-141. (10.1530/jrf.0.0210133) 4983966

[36] Nunn CL. 1999 The evolution of exaggerated sexual swellings in primates and the graded-signal hypothesis. Anim. Behav. **58**, 229-246. (10.1006/anbe.1999.1159) 10458874

[37] Zinner D, Deschner T. 2000 Sexual swellings in female hamadryas baboons after male take-overs: ‘deceptive’ swellings as a possible female counter-strategy against infanticide. Am. J. Primatol. **52**, 157-168. (10.1002/1098-2345(200012)52:4<157::AID-AJP1>3.0.CO;2-L) 11132110

[38] Vandenbergh JG. 1967 Effect of the presence of a male on the sexual maturation of female mice. Endocrinology **81**, 345-349. (10.1210/endo-81-2-345) 4952008

[39] Lu A, Feder JA, Snyder-Mackler N, Bergman TJ, Beehner JC. 2021 Male-mediated maturation in wild geladas. Curr. Biol. **31**, 214-219.e2. (10.1016/j.cub.2020.10.003) 33157017

[40] Bruce HM. 1959 An exteroceptive block to pregnancy in the mouse. Nature **184**, 105. (10.1038/184105a0) 13805128

[41] Roberts EK, Lu A, Bergman TJ, Beehner JC. 2012 A Bruce effect in wild geladas. Science **335**, 1222-1225. (10.1126/science.1213600) 22362878

[42] Tinsley Johnson E, Snyder-Mackler N, Lu A, Bergman TJ, Beehner JC. 2018 Social and ecological drivers of reproductive seasonality in geladas. Behav. Ecol. **29**, 574-588. (10.1093/beheco/ary008) 29769792 PMC5946938

[43] Palombit RA, Cheney DL, Seyfarth RM. 2001 Female–female competition for male ‘friends’ in wild chacma baboons (*Papio cynocephalus ursinus*). Anim. Behav. **61**, 1159-1171. (10.1006/anbe.2000.1690)

[44] Cheney DL, Silk JB, Seyfarth RM. 2012 Evidence for intrasexual selection in wild female baboons. Anim. Behav. **84**, 21-27. (10.1016/j.anbehav.2012.03.010) 25558080 PMC4280838

[45] Clutton-Brock T, Huchard E. 2013 Social competition and its consequences in female mammals. J. Zool. **289**, 151-171. (10.1111/jzo.12023)

[46] Baniel A, Cowlishaw G, Huchard E. 2016 Stability and strength of male-female associations in a promiscuous primate society. Behav. Ecol. Sociobiol. **70**, 761-775. (10.1007/s00265-016-2100-8)

[47] Huchard E, Alvergne A, Féjan D, Knapp LA, Cowlishaw G, Raymond M. 2010 More than friends? Behavioural and genetic aspects of heterosexual associations in wild chacma baboons. Behav. Ecol. Sociobiol. **64**, 769-781. (10.1007/s00265-009-0894-3)

[48] Carter AJ, Baniel A, Cowlishaw G, Huchard E. 2020 Baboon thanatology: responses of filial and non-filial group members to infants' corpses. R. Soc. Open Sci. **7**, 192206. (10.1098/rsos.192206) 32269818 PMC7137963

[49] Carter AJ, Torrents Ticó M, Cowlishaw G. 2016 Sequential phenotypic constraints on social information use in wild baboons. eLife **5**, e13125. (10.7554/eLife.13125) 27067236 PMC4829417

[50] Gesquiere LR, Wango EO, Alberts SC, Altmann J. 2007 Mechanisms of sexual selection: sexual swellings and estrogen concentrations as fertility indicators and cues for male consort decisions in wild baboons. Horm. Behav. **51**, 114-125. (10.1016/j.yhbeh.2006.08.010) 17027007

[51] Darmis F, Huchard É, Cowlishaw G, Carter AJ. 2023 Data from: Cycle length flexibility: is the duration of sexual receptivity associated with changes in social pressures? Dryad Digital Repository. (10.5061/dryad.r4xgxd2h3) PMC10685116 38034125

[52] Nakagawa S, Schielzeth H. 2010 Repeatability for Gaussian and non-Gaussian data: a practical guide for biologists. Biol. Rev. **85**, 935-956. (10.1111/j.1469-185X.2010.00141.x) 20569253

[53] Stoffel MA, Nakagawa S, Schielzeth H. 2017 rptR: repeatability estimation and variance decomposition by generalized linear mixed-effects models. Methods Ecol. Evol. **8**, 1639-1644. (10.1111/2041-210X.12797)

[54] Bates D, Mächler M, Bolker B, Walker S. 2015 Fitting linear mixed-effects models using lme4. J. Stat. Soft. **67**, 1–48. (10.18637/jss.v067.i01)

[55] Barr DJ, Levy R, Scheepers C, Tily HJ. 2013 Random effects structure for confirmatory hypothesis testing: keep it maximal. J. Mem. Lang. **68**, 255-278. (10.1016/j.jml.2012.11.001) PMC3881361 24403724

[56] Mazerolle MJ. 2020 AICcmodavg: model selection and multimodel inference based on (Q)AIC(c). See https://mirrors.cloud.tencent.com/CRAN/web/packages/AICcmodavg/vignettes/AICcmodavg.pdf.

[57] Bates D, Kliegl R, Vasishth S, Baayen H. 2018 Parsimonious mixed models. *arXiv.* (10.48550/arXiv.1506.04967)

[58] Matuschek H, Kliegl R, Vasishth S, Baayen H, Bates D. 2017 Balancing Type I error and power in linear mixed models. J. Mem. Lang. **94**, 305-315. (10.1016/j.jml.2017.01.001)

[59] Lüdecke D, Ben-Shachar MS, Patil I, Waggoner P, Makowski D. 2021 performance: an R package for assessment, comparison and testing of statistical models. J. Open Source Softw. **6**, 3139. (10.21105/joss.03139)

[60] James G, Witten D, Hastie T, Tibshirani R. 2013 An introduction to statistical learning. New York, NY: Springer. (10.1007/978-1-4614-7138-7)

[61] Francoeur RB. 2013 Could sequential residual centering resolve low sensitivity in moderated regression? Simulations and cancer symptom clusters. Open J. Statistics **3**, 24-44. (10.4236/ojs.2013.36A004)

[62] Viau P, Rodini DC, Sobral G, Martins GS, Morato RG, de Oliveira CA. 2020 Puberty and oestral cycle length in captive female jaguars *Panthera onca*. Conserv. Physiol. **8**, coaa052. (10.1093/conphys/coaa052) 32577289 PMC7296220

[63] Shea AA, Vitzthum VJ. 2020 The extent and causes of natural variation in menstrual cycles: integrating empirically-based models of ovarian cycling into research on women's health. Drug Discovery Today: Dis. Models **32**, 41-49. (10.1016/j.ddmod.2020.11.002)

[64] Mihm M, Gangooly S, Muttukrishna S. 2011 The normal menstrual cycle in women. Anim. Reprod. Sci. **124**, 229-236. (10.1016/j.anireprosci.2010.08.030) 20869180

[65] Alvergne A, Högqvist Tabor V. 2018 Is female health cyclical? Evolutionary perspectives on menstruation. Trends Ecol. Evol. **33**, 399-414. (10.1016/j.tree.2018.03.006) 29778270

[66] Markham AC, Gesquiere LR, Alberts SC, Altmann J. 2015 Optimal group size in a highly social mammal. Proc. Natl Acad. Sci. USA **112**, 14 882-14 887. (10.1073/pnas.1517794112) 26504236 PMC4672815

[67] Alberts SC, Sapolsky RM, Altmann J. 1992 Behavioral, endocrine, and immunological correlates of immigration by an aggressive male into a natural primate group. Horm. Behav. **26**, 167-178. (10.1016/0018-506x(92)90040-3) 1612563

[68] Stevenson PR, Castellanos MC. 2000 Feeding rates and daily path range of the Colombian woolly monkeys as evidence for between- and within-group competition. Folia Primatol. (Basel) **71**, 399-408. (10.1159/000052737) 11155028

[69] Darmis F, Huchard É, Cowlishaw G, Carter AJ. 2023 Cycle length flexibility: is the duration of sexual receptivity associated with changes in social pressures? Figshare. (10.6084/m9.figshare.c.6948913) PMC10685116 38034125

